# Exploration of the cortical pathophysiology underlying visual disturbances in schizophrenia comorbid with depressive disorder—An evidence from mouse model

**DOI:** 10.1002/brb3.2113

**Published:** 2021-03-17

**Authors:** Jian Liu, Lidan Zheng, Tao Fang, Ranli Li, Xiaoyan Ma, Yun Sun, Lina Wang, Hongjun Tian, Deguo Jiang, Chuanjun Zhuo

**Affiliations:** ^1^ Laboratory of Psychiatric‐Neuroimaging‐Genetic and Cor‐morbidity (PNGC_Lab) Tianjin Anding Hospital Mental Health Centre of Tianjin Affiliated Teaching Hospital of Tianjin Medical University Tianjin China; ^2^ Department of Psychiatry Wenzhou Seventh Peoples Hospital Wenzhou China; ^3^ Key Laboratory of Real‐Time Tracing of Brain Circuits of Neurology and Psychiatry (RTBNB_Lab) Tianjin Fourth Centre Hospital Tianjin Medical University Affiliated Tianjin Fourth Centre Hospital Nankai University Affiliated Fourth Hospital Tianjin China

**Keywords:** animal model, depression, schizophrenia, visual cortex, visual hallucination

## Abstract

**Introduction:**

Patients with schizophrenia frequently present with visual disturbances including hallucination, and this symptom is particularly prevalent in individuals with comorbid depressive disorders. Currently, little is known about the neurobiological mechanisms of such psychiatric symptoms, and few explanations for the co‐occurrence of schizophrenia, depression, and visual disturbances are available.

**Methods:**

In this study, we generated a mouse schizophrenia model in which depressive symptoms were also induced. We adopted in vivo two‐photon calcium imaging and ex vivo electrophysiological recording of the primary visual cortex to reveal the synaptic transmission and neural activity in the mouse schizophrenia model.

**Results:**

In vivo two‐photon calcium imaging and ex vivo electrophysiological recording of the primary visual cortex revealed impaired synaptic transmission and abnormal neural activity in the schizophrenia model, but not in the depression model. These functional deficits were most prominent in the combined schizophrenia and depression model.

**Conclusion:**

Overall, our data support a mechanism by which the visual cortex plays a role in visual disturbances in schizophrenia.

## Graphical abstract

In our mouse model of schizophrenia, the hyperactivation of cortical neurons occurred without exposure to sensory stimuli, as supported by in vivo and ex vivo recordings. This elevated spontaneous activity was attributable mainly to a greater frequency, but not amplitude, of calcium activity spikes, indicating the potentiation of presynaptic transmission. Notably, comorbidity with depression further aggravated the cortical synapse pathology. These pathophysiological alterations in the visual cortex provide a possible explanation for the occurrence of visual hallucination in patients with schizophrenia.

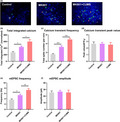

## 1 INTRODUCTION

Schizophrenia is a psychotic disorder with an approximately 0.5% lifetime prevalence (Kahn et al., 2015; Simeone et al., 2015). Major clinical manifestations of schizophrenia include delusion, visual disturbances including hallucination, and disorganized speech (Soares‐Weiser et al., 2015). Among those with psychotic syndromes, hallucinations frequently present in auditory‐verbal, auditory, and/or visual forms and are core features of the disease (Waters and Fernyhough, 2017; Hugdahl & Sommer, 2018). A long period of untreated hallucination is a strong predictor of poor prognosis among patients with schizophrenia (Stentebjerg‐Olesen et al., 2016). Neuropathological studies examining schizophrenia‐associated hallucination are thus important to improve patient outcomes. Knowledge of the neurobiological underpinnings of different forms of hallucination is limited, due in part to the lack of effective animal models.

Human imaging studies have shown that hallucinations are frequently associated with abnormal neural activity in primary and secondary sensory regions (Brown and Thompson, 2010). Among cortical areas, the auditory cortex and prefrontal cortex (PFC) have been found to be involved in the auditory‐verbal hallucinations of patients with schizophrenia (Huang et al., 2019). In those patients, functional imaging data also suggested activation of the visual cortex in association with structural and connectivity changes (Waters et al., 2014). Based on such clinical evidence and considering the findings of previous studies, investigations were undertaken and revealed that the N‐methyl‐D‐aspartate (NMDA) receptor antagonist ketamine could induce schizophrenia symptoms, such as visual hallucination (Incrocci et al., 2018), and that the NMDA receptor antagonist MK801 could induce hallucination (Shokry et al., 2019).

The mimicking of visual disturbances including hallucination in animal models is a major challenge in schizophrenia research, due mainly to rodents’ lack of self‐report ability and the infeasibility of quantifying abnormal behavior during hallucination episodes. However, due to the abnormal neural activity of visual cortical regions in human patients with functional disturbances, our study raised the possibility such pathological phenotypes in patients can be partially mimicked and verified using relevant animal models. Chronic treatment with MK801 induced more spontaneous activity in the visual cortex, like that reflected in clinical imaging data from patients. Based on this similarity, we have confidence that the cortical circuitry and molecular mechanisms underlying this effect can be further examined using our mouse model, and that the output from animal studies will aid the identification of new diagnostic and treatment targets for schizophrenia‐related visual disturbances. We thus expanded the use of the MK801‐treated mouse model from mere replication of the sensorimotor symptoms of schizophrenia to mimicking of the pathology of visual disturbances. Although this model does not necessarily reflect all features of visual hallucination in human patients, it may be a valuable tool in efforts to further advance the study of schizophrenia.

In this study, we generated a mouse model of schizophrenia using MK801. Compared with healthy and depressed animals, the schizophrenic mice showed elevated neural activity in the primary visual cortex (V1) in the absence of external visual stimulus, detected by in vivo two‐photon calcium imaging and ex vivo electrophysiological recording. Moreover, schizophrenic mice comorbid with depression presented worsening cortical neural function. Collectively, our data suggest the cortical mechanism by which schizophrenia distorts the normal functioning of the visual pathway, probably contributing to visual disturbances phenotypes.

## 2 MATERIALS AND METHODS

### 2.1 Animals and experimental design

Four‐week‐old male C57BL/6J mice were used in all experiments unless otherwise specified. The mice were kept in ventilated animal housing with food and water available ad libitum. To generate the schizophrenia model, the mice were given intraperitoneal injections of 1 mg/kg MK801 daily for 14 consecutive days. To generate the depression model, the mice were subjected to CUMS following a previously published protocol (Leng et al., 2018). In brief, the mice were housed singly and subjected to an established battery of negative stimuli (food or water deprivation, restraint stress, cage tilt, overnight illumination, and forced swimming) implemented in random order for 14 days. After the conclusion of the pharmacological or behavioral intervention, behavioral assays were performed for model validation, followed by two‐photon imaging or patch clamp recording.

### 2.2 Behavioral assays

#### 2.2.1 PPI assay

This assay was performed in a sound‐attenuated chamber using previously published paradigms (Koukouli et al., 2017; Nielsen et al., 2017) to test the sensorimotor function of all mice. In brief, the mice were first acclimated for 5 min with white noise (62 dB). Then, 30 startle pulses (105 dB) were applied consecutively with a 10‐s intertrial interval in the habituation stage. During the testing session, the prepulse was set at 15 dB above background (i.e., 77 dB), and the inter‐stimulus interval was 100 ms. Each test session consisted of eight trials, and pulse magnitudes were recorded. PPI was calculated as follows: PPI = (pulse‐only score – prepulse with pulse score) / pulse‐only score × 100%.

#### 2.2.2 Forced swimming test

Based on an established protocol (Yankelevitch‐Yahav et al., 2015), each mouse was placed in a cylindrical water tube. The time spent immobile was determined from a video recording.

#### 2.2.3 Sucrose preference test

This assay was conducted following a previously published protocol (Su et al., 2017). The mice were deprived of food and water for 24 hr and then provided with two bottles containing clean water and a 1% sucrose solution, respectively. Fluid consumption during 1 hr was quantified to evaluate anhedonic behavior.

### 2.3 Two‐photon calcium recording

Neuronal activity in the PFC was measured using a slight modification of a previously reported method (Vasalauskaite et al., 2019) except the specific coordination of visual cortex for making transcranial imaging window. In brief, each anesthetized mouse was fixed to a stereotaxic stage, and a chronic cranial window was created. Then, 200 nl of the AAV 2/9‐syn‐GCaMP6s (2 × 10^13^ genome copies/mL; University of Pennsylvania Vector Core Facility) was injected bilaterally into the prelimbic cortex at + 2.8 ± 0.5 mm from bregma. The imaging window was covered with a circular coverslip, and the skull was sealed using dental cement. A customized steel bar was embedded into the skull for fixation of the mouse's head during imaging.

Two‐photon in vivo imaging was performed using standard protocols (Förster et al., 2017) with a two‐photon microscope (LSM780; Zeiss, Germany) with a 16×, 0.8 numerical aperture water‐immersion objective. Using an excitation wavelength of 950 nm, time‐series images were recorded for 150 s at 1.96 Hz. The captured images were analyzed using ImageJ software (National Institutes of Health, Bethesda, MD, USA). Regions of interest were selected manually using ImageJ software with the FIJI plug‐in package. Calcium transients were then detected and normalized.

### 2.4 Patch clamp recording

We used electrophysiological recordings to measure cortical PN activity, following a previously reported method (Vélez‐Fort et al., 2018). The mice were anesthetized deeply with isoflurane and decapitated. Each brain was removed and sectioned into 250‐μm‐thick slices, which were incubated in 30°C artificial cerebrospinal fluid (aCSF) containing 194 mM sucrose, 20 mM NaCl, 4.4 mM KCl, 2 mM CaCl_2_, 1 mM MgCl_2_, 1.2 mM NaH_2_PO_4_, 10 mM glucose, and 26 mM NaHCO_3_. After 1 hr of recovery, the brain slices were transferred to a recording chamber infused with oxygenated aCSF. A recording electrode prepared from a borosilicate glass capillary tube with 3–5 MΩ resistance was used. L2/3 PNs were patched using a pipette containing a solution of 135 mM K‐Gluc, 5 mM NaCl, 2 mM MgCl_2_, 10 mM HEPES, 0.6 mM EGTA, 4 mM Na_2_ATP, and 0.4 mM Na_2_GTP. The cells were voltage clamped at –70 mV for mEPSC recording in the presence of tetrodotoxin. The signals were digitized at 10 kHz, filtered at 3 kHz using an amplifier (Multiclamp 700B; Molecular Devices), and analyzed using Clampfit software (ver. 10.3).

### 2.5 Statistical analysis

All data are presented as means ± standard errors of the mean unless otherwise specified. The data were compared using one‐way analysis of variance with Tukey's post hoc test. GraphPad Prism software (ver. 8.0) was used for data analysis and figure creation.

## 3 RESULTS

### 3.1 Schizophrenic mice had abnormally elevated spontaneous calcium activity

**FIGURE 1:**
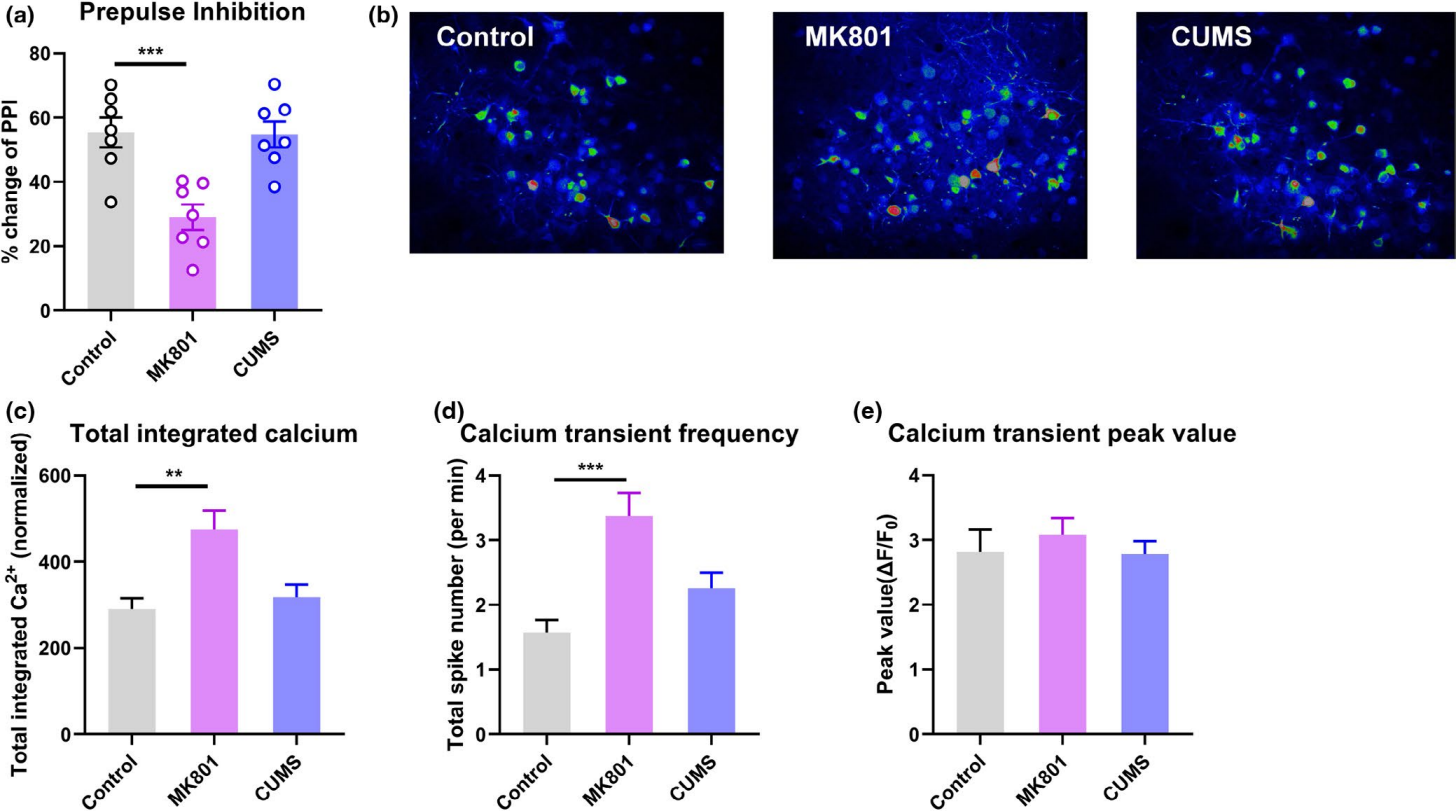
Schizophrenic mice showed more spontaneous calcium activity in the primary visual cortex. (a) Changes in the prepulse inhibition ratio among control, MK801‐treated, and CUMS‐exposed mice. (b) Representative pseudo‐color images showing calcium activity in the visual cortex. (c) Integrated calcium activity (in ΔF/F_0_) in the visual cortex during the recording period. (d) Average numbers of calcium transient spikes per minute (frequency). (e) Average peak values of individual calcium spikes. **p < .05, ***p < .001, one‐way ANOVA with Tukey's post hoc comparison. *N* = 7 mice per group

We generated a mouse schizophrenia model using MK801 injections and performed a prepulse inhibition (PPI) assay, a classic means of identifying sensorimotor gating deficits in schizophrenia (Schwabe and Krauss, 2018). We identified prominent deficits, reflected by PPI ratios, in MK801‐treated animals (Figure 1a), but not in depressed mice following exposure to chronic unpredictable mild stress (CUMS), suggesting that sensorimotor gating impairment is specific to schizophrenia.

Next, we used two‐photon in vivo imaging to record neuronal calcium activity in V1 after adeno‐associated virus (AAV)‐aided transfection of the genetically coded fluorescent calcium indicator GCaMP6s. In awake mice in the schizophrenia group in a dark environment, we found significantly elevated levels of spontaneous calcium transients in layer 2/3 pyramidal neurons (L2/3 PNs) in V1 (Figure 1b and c); healthy control and depressed mice showed less calcium activity. Further data analysis showed that this abnormal increase in calcium activity was attributable mainly to a greater frequency of activity spikes (Figure 1d), while the amplitude of each spike remained largely unchanged (Figure 1e). These data demonstrated the occurrence of potentiated cortical activity in V1 even with minimal visual stimulation.

### 3.2 Abnormally elevated synaptic transmission occurred in schizophrenic mice

**FIGURE 2:**
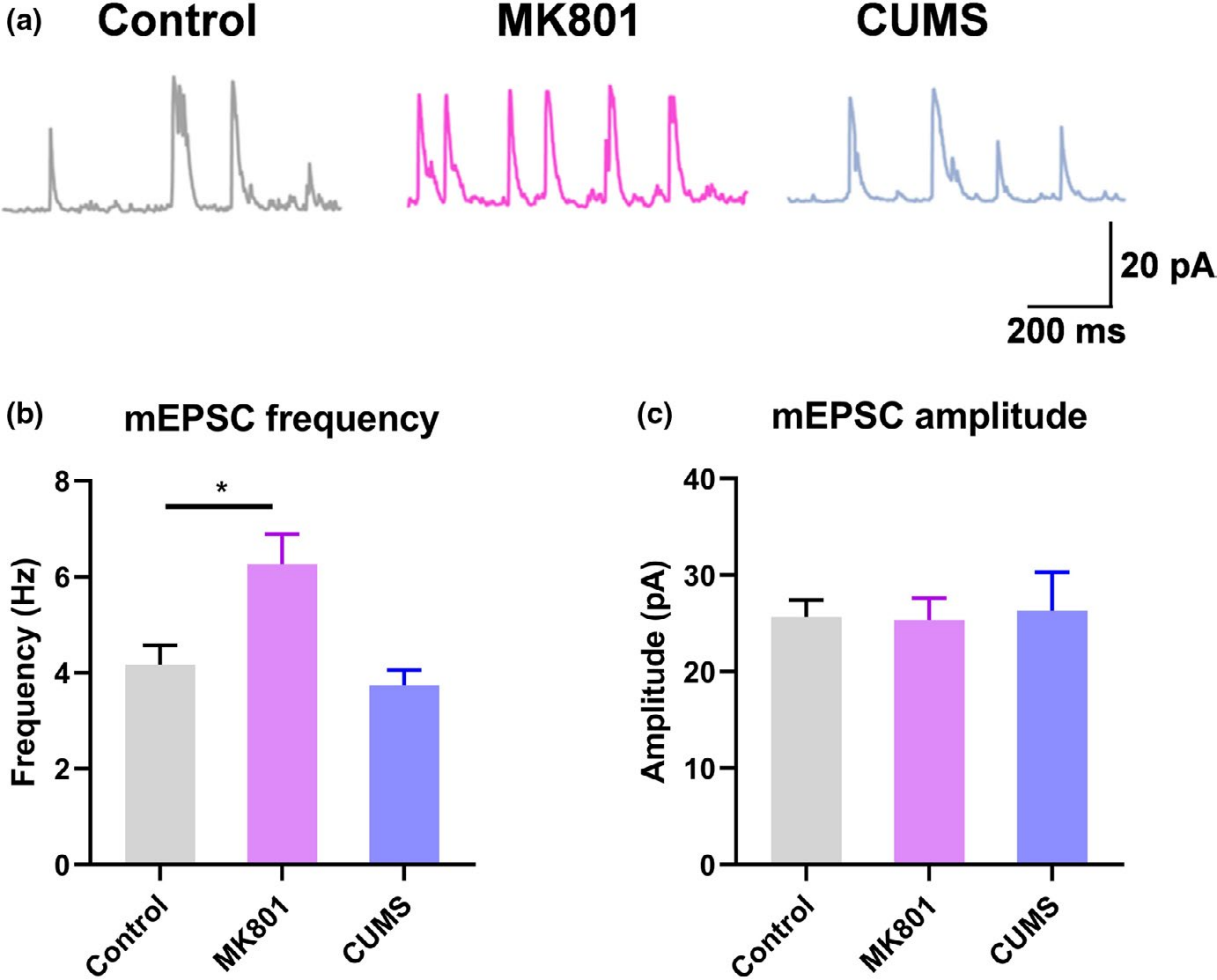
Potentiated synaptic transmission in the visual cortex in schizophrenic mice. (a) Representative traces of miniature excitatory postsynaptic currents (mEPSCs) in control, schizophrenic, and depressed mice. (b) Frequencies (in Hz) of mEPSCs. (c) Amplitudes (in pA) of mEPSCs. *p < .05, one‐way ANOVA with Tukey's post hoc comparison. *N* = 25 neurons (from 4 mice) per group

Next, we studied the synaptic function of L2/3 PNs in V1. By applying patch clamp recording to ex vivo brain slices, we recorded miniature excitatory postsynaptic currents (mEPSCs). We found potentiated mEPSCs in the excitatory neurons (Figure 2a). Specifically, schizophrenic mice presented a greater frequency of mEPSCs occurring without stimulation (Figure 2b), with the amplitude of most mEPSCs remaining unchanged (Figure 2c). These observations are in agreement with those from the in vivo calcium recording, and indicate the increased presynaptic release of excitatory neurotransmitters in the schizophrenia model, with no effect on postsynaptic excitability. No such mEPSC potentiation was observed in depressed mice, which presented phenotypes similar to those of the control mice. Thus, the schizophrenia mouse model features remarkably elevated neuronal activity in V1, a manifestation of a unique neuropathological feature of the disease.

### 3.3 Deterioration of neural function was observed in a comorbid model

**FIGURE 3:**
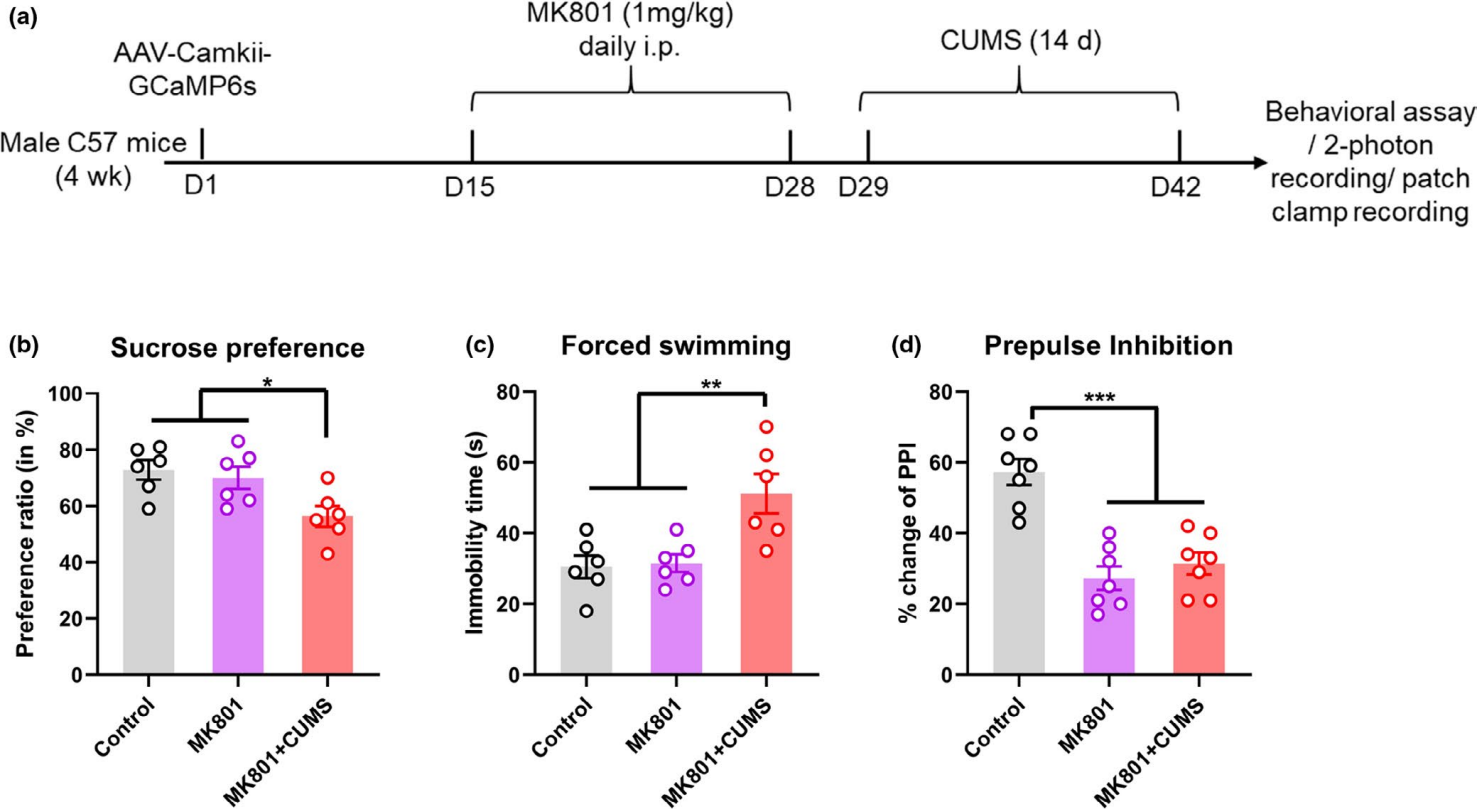
Generation of the mouse model of schizophrenia comorbid with depression. (a) Experimental timelines for model preparation, behavioral tests depicted in this figure, and functional assays depicted in Figure 4. (b) Anhedonia phenotypes of control mice, mice with schizophrenia, and mice with schizophrenia and depression, determined using a sucrose preference test. (c) Immobility times (reflecting despair) in the forced swimming test. *p < .05, **p < .01, ***p < .001, one‐way ANOVA with Tukey's post hoc comparison. *N* = 6 animals per group

There is a high prevalence (20%~60% depending on the disease stage) of depressive disorders in patients with schizophrenia (Upthegrove et al., 2017), it is thus necessary to delineate the effect of depressive disorders on neuropathology of schizophrenia. Here, we investigated whether the observed functional disruption in V1 also existed in a comorbid model. We generated a cohort of mice with schizophrenia‐like and depressive behaviors using a CUMS intervention following MK801 injection (Figure 3a). Behavioral tests revealed anhedonia and depressive phenotypes in these mice compared with mice treated with MK801 alone (Figure 3b and c). The mice also showed sensorimotor gating deficits in a PPI paradigm (Figure 3d), suggesting the presentation of typical schizophrenia‐like behaviors.

**FIGURE 4:**
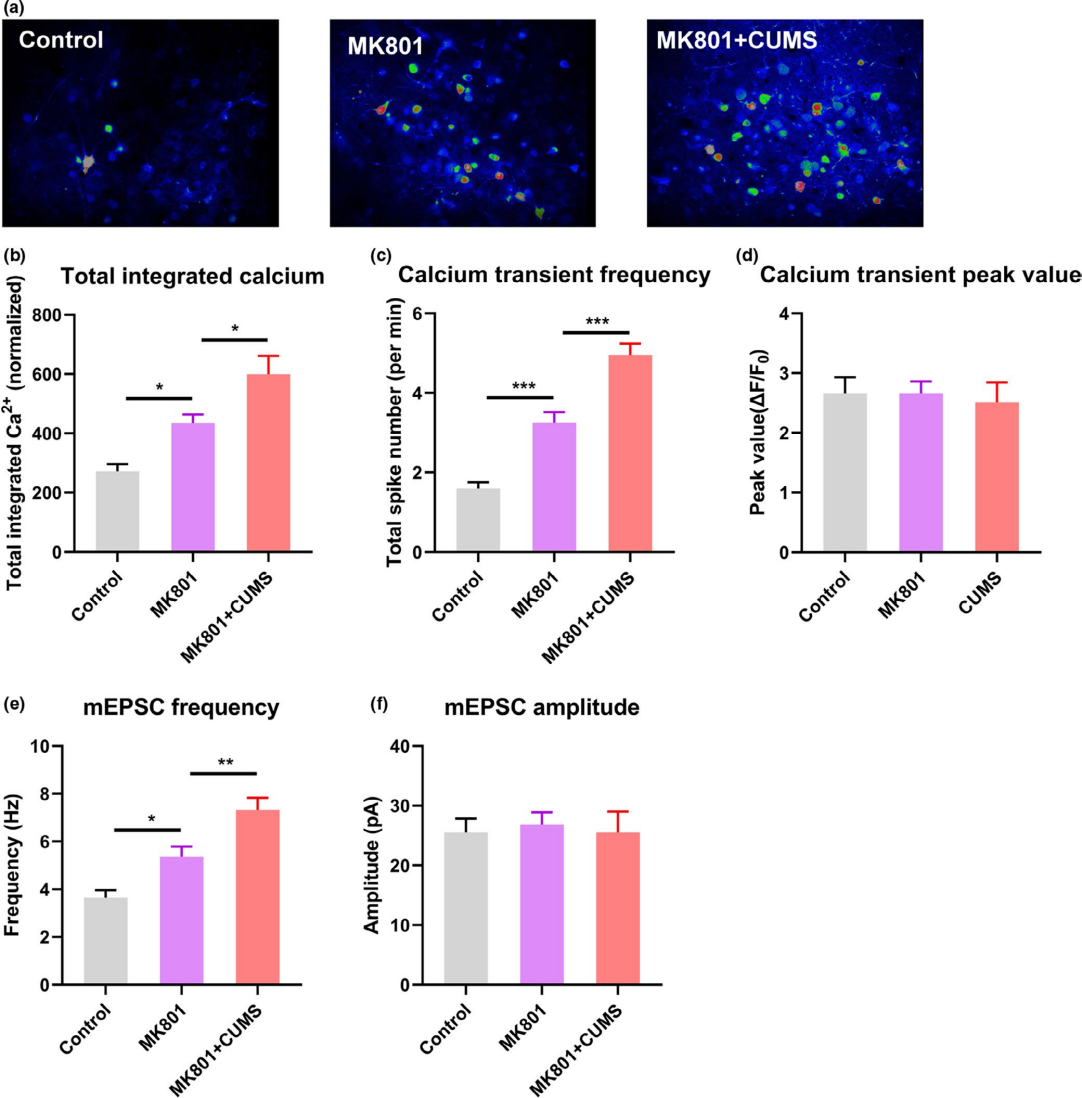
Schizophrenia comorbid with depression aggravated the impairment of visual cortical function. (a) Representative pseudo‐color images showing neuronal activity in the visual cortex in the control, schizophrenia, and schizophrenia with depression groups. (b) Total integrated calcium activity of L2/3 PNs in V1. (c) Average numbers of calcium transient spikes. (d) Peak calcium transient values. (e) Frequencies of mEPSCs in the three groups, determined using ex vivo electrophysiological recording. (f) Amplitudes of mEPSCs. *p <.05, **p < .01, ***p < .001, one‐way ANOVA with Tukey's post hoc comparison. *N* = 30 neurons (from 5 animals) per group

Using two‐photon calcium imaging, we recorded the same type of spontaneous neuronal activity in V1 as observed in the schizophrenia model. The calcium activity level was higher, with a greater frequency but unchanged amplitude of calcium transients, in the comorbid mice than in MK801‐treated mice (Figure 4a–d). In agreement with the in vivo data, electrophysiological recordings from brain slices suggested a greater frequency of mEPSCs in L2/3 PNs in V1, with unchanged amplitudes (Figure 4e and f). Collectively, these data indicate the hyperactivation of spontaneous activity in the visual cortex in schizophrenic mice with depressive behaviors.

## 4 DISCUSSION

In our mouse model of schizophrenia, the hyperactivation of cortical neurons occurred without exposure to sensory stimuli, as supported by in vivo and ex vivo recordings. This elevated spontaneous activity was attributable mainly to a greater frequency, but not amplitude, of calcium activity spikes, indicating the potentiation of presynaptic transmission. Notably, comorbidity with depression further aggravated the cortical synapse pathology. These pathophysiological alterations in the visual cortex provide a possible explanation for the occurrence of visual disturbances including hallucination in patients with schizophrenia.

As a major higher processing center for visual input, V1 is highly interconnected with other cortical and subcortical nuclei for further interpretation of visual signals and visually related behaviors. For example, neurons in the visual cortex are known to project to medial prefrontal regions (Kim et al., 2003), and bidirectional connections exist between V1 and the secondary motor cortex for predictive visual signals (Leinweber et al., 2017). Thus, the abnormal excitation of neurons in the visual cortex may affect the motor, emotional, and other systems, leading to the misinterpretation of visual signals. Our in vivo and ex vivo recording results strongly support the occurrence of abnormally high levels of spontaneous activity in the visual cortex, even without the presentation of external visual stimuli, in our schizophrenia mouse model. Such hyperactivation in V1 thus leads to distorted activation of the brain network, leading to the induction of visual function distrubances.

The schizophrenic mice complicated with depressive symptoms presented further aggravation of cortical hyperactivation. Clinical data suggest that auditory hallucination is a predictive factor for depressive symptoms in patients with schizophrenia (Onwuameze et al., 2016). Other data reveal associations between hallucination and depressive symptoms in such patients (Chiang et al., 2018). Thus, hallucination and depressive symptoms may be underlain by common neuropathological mechanisms, which probably contributes to dysfunction in the visual cortex. The possibility that visual disturbances is aggravated in schizophrenic individuals with depression has clinical implications. Since the comorbid rate of depression ranges between 20%~60% in schizophrenia individuals (Upthegrove et al., 2017). Clinicians thus should monitor such patients carefully to achieve timely intervention. In principle, our data provide more hints for clinical diagnosis and intervention of schizophrenia: Firstly, it is necessary to perform routine functional imaging study on schizophrenia patients, especially those presenting depressive behaviors. Secondly, the sign of abnormal activity in visual region may indicate ongoing depression syndromes in schizophrenia patients. Lastly, neuromodulation approaches such as transcranial magnetic stimulation (TMS) that can effectively inhibit cortical neural activity may help to ameliorate at least visual disturbances in those patients.

How schizophrenia affects visual cortical function is an intriguing question. Using our animal model, this effect can be examined via MK801 blockage of NMDA receptors. Although it seems paradoxical that a glutamate receptor antagonist leads to the hyperactivation of cortical neurons, MK801 likely exerts its effects by suppressing GABAergic inhibitory neurons in the cortex, leading to the disinhibition of L2/3 PNs. This hypothesis is supported by the antidepressant role of ketamine, an alternative NMDA receptor antagonist. Recent studies suggest that ketamine exerts its effects by inhibiting NMDA receptors on GABAergic interneurons, thereby increasing the activity of PNs in prefrontal regions (Widman and McMahon, 2018; Zanos & Gould, 2018). In a schizophrenia models, the effects of NMDA receptor antagonists on interneurons led to disruption of the excitatory–inhibitory balance (Cadinu et al., 2018). Further investigation of the inhibitory circuits in the visual cortex in schizophrenia animal models and in patients may help us to better identify the underlying pathological mechanisms.

In this study, we obtained neuronal recording data demonstrating cortical hyperactivation in schizophrenic mice. Since abnormal cortical activity is frequently observed in human patients suffering from visual disturbances including hallucination, we generated a model that can help to study the neural mechanism due to the similarity of cortical hyperactivation between rodents and humans. In addition, much severe cortical dysfunction was observed in schizophrenic mice with depressive symptoms, providing new insight into the neuropathology underlying comorbid psychiatric diseases.

## 5 ETHICS APPROVAL

The protocol was approved by the local ethics committee.

## CONFLICT OF INTEREST

The authors declare that they have no competing interest.

## AUTHOR CONTRIBUTIONS

CJZ, CHZ, and HJT designed and undertook the experiments; QCL, RLL, YS, LNW, TF, and CHZ made the animal model; CHZ and TF performed the statistical analysis; CJZ wrote the paper; CJZ and CHZ revised the manuscript. All authors contributed to this work, from animal model establishment to visual cortex activity observation, and approved the publication of this manuscript.

### PEER REVIEW

The peer review history for this article is available at https://publons.com/publon/10.1002/brb3.2113.

## Data Availability

The data that support the findings of this study are available from the corresponding author upon reasonable request.
